# On Second Thought

**DOI:** 10.1093/asjof/ojz033

**Published:** 2020-01-14

**Authors:** Lorne K Rosenfield

“Procedures come alive in the tinkering, fussing hands of their operators, who navigate seemingly insurmountable challenges...What’s transmitted—manually, individually, artisanally—to the next generation of surgeons is a process rather than a product, a skill rather than a pill. An apprentice practices the procedure over and over as if taking lessons in an immensely complicated musical instrument; the teacher looks for the sharpness, the fettle that comes with a hundred attempts...Procedures are typically created, nurtured, and perfected in a few hospitals, and they spread as the apprentices gain mastery, move to new places, and promulgate their know-how: see one, do one, teach one.” 

Siddhartha Mukherjee, MD^1^

One could sum up Dr. Mukherjee’s poetic description of a surgeon’s procedural tinkering in just two words: “Learning Curve.” That is, when a particular surgical technique is first presented, in whatever venue or format, it can easily beguile one into thinking that it is perfected. 2 Instead, any approach is actually in a constant state of refinement and evolution, as knowledge, technology, and ability advance. Yet, a scientific paper, book chapter, meeting lecture, or technical video delivers, at best, a description that is valid only at the time of its publication or presentation: the surgical technique’s “present.” The offering is more like a snapshot, forever frozen in the moment, devoid of the narrative as to both *how* the surgical technique came about initially, or *what* about it may still be evolving: the surgical technique’s “past” and “future.” ^3^ And it is the melding of all three of these phases of a technique’s evolution that describes a powerfully elucidating arc, the surgeon’s “learning curve.”

Steve Jobs spoke of how one can only connect the seminal “dots” of one’s past, *after* the fact.^4^ Well, those same dots represent the surgical high and low moments that when connected, plot and portray one’s learning curve. So, if one were to ask a surgeon about these same seminal dots—the origins of a particular principle, technique, or strategy—we would be treated to their insights about the “tinkering” they did as they were formulating their original ideas.

And so, enter a new, thought-provoking section of *ASJ Open Forum* called *Second Thoughts on First Thoughts*. Each quarter, I will interview a colleague about a surgical topic about which they are passionate and for which they are renowned. But the elucidating twist in this endeavor is that I will be taking a deep dive into the evolution of their approach: the past choke points, the steps in technique that initially failed and how they led to a better solution; the present high points, the strategies that now deliver consistently better results; and the future inflection points, the postoperative complications and aesthetic results that they are still working on preventing or realizing, respectively. Ultimately, we will all come away enriched with the underlying principles that were birthed by this process and which guided the surgeon as they ascended their learning curve. And in turn, this experience will hopefully spur us all forward as we summit the slopes of our own learning curves!

So, keep a lookout for the first installment of this inspiring new addition to our already uniquely impactful journal!
